# Risk factors associated with colorectal adenomas and the formulation of prediction models

**DOI:** 10.3389/fonc.2026.1894239

**Published:** 2026-10-12

**Authors:** Haoming Yu, Zongyu Qiu, Wenyao Sui, Shengyu Han, Jihong Li, Wenhua Lan, Xuan Sun, Yuhua Chi

**Affiliations:** 1 Department of General Medicine, Affiliated Hospital of Shandong Second Medical University, Weifang, Shandong, China; 2 College of Clinical Medicine, Shandong Second Medical University, Weifang, Shandong, China

**Keywords:** colorectal adenoma, machine learning algorithms, metabolic markers, risk prediction model, triglyceride-glucose index

## Abstract

**Importance:**

Colorectal adenomas (CRAs) are the primary precancerous lesions in the development of colorectal cancer; establishing a simple, cost-effective and non-invasive tool for distinguishing adenomatous from non-neoplastic polyps in patients already scheduled for or undergoing endoscopy is of great significance for risk stratification.

**Objective:**

To investigate the association between peripheral blood metabolic and inflammatory markers and the risk of colorectal adenomas, and to develop a personalised risk prediction model for distinguishing adenomatous from non-neoplastic polyps in patients already scheduled for or undergoing endoscopy.

**Design, setting, and participants:**

This study was a single-centre retrospective case-control study conducted at the Affiliated Hospital of Shandong Second Medical University. The study included 2,437 patients with colorectal polyps who underwent endoscopic resection between January 2020 and December 2025.

**Exposures:**

Demographic characteristics, lifestyle factors and peripheral blood biochemical markers, with a particular focus on the triglyceride-to-glucose (TyG) ratio and markers of systemic inflammation.

**Main outcomes and measure:**

Patients with colorectal adenomas confirmed by histopathology. Model performance was assessed using the area under the receiver operating characteristic curve (AUC), calibration curves and decision curve analysis (DCA).

**Results:**

The study included 2,437 participants (mean age 58.57 years; 1,323 men [54.29%]), patients in the adenoma group (n=1,584) were significantly older and had a higher proportion of men compared with the control group (n=853). Multivariate logistic regression analysis revealed that male sex (OR, 1.621; 95% CI, 1.269-2.074), age ≥56.5 years (OR, 1.868; 95% CI, 1.515-2.205) and a TyG index ≥8.562 (OR, 1.271; 95% CI, 1.015-1.590) were independent risk factors for the development of colorectal adenomas. The AUC of this predictive model was 0.631 and 0.607 in the training and validation sets, respectively, indicates moderate discriminatory power. The model is well calibrated; analysis of the decision curve shows that, across a range of threshold probabilities, the model yields a positive net benefit compared with strategies involving either universal treatment or universal non-treatment.

**Conclusions and relevance:**

The findings suggest that male gender, advanced age and an elevated TyG index are independently associated with the risk of colorectal adenomas. The non-invasive nomogram developed in this study provides a simple risk stratification tool to aid in distinguishing adenomatous from non-neoplastic polyps in patients already scheduled for or undergoing endoscopy.

## 1 Introduction

Colorectal adenomas (CRA) are the primary precancerous lesions in the development of colorectal cancer (1). Colorectal cancer (CRC) is a malignant tumour of the digestive system with relatively high incidence and mortality rates worldwide, and its prevention and treatment have become a key focus for public health systems across the globe. Driven by the advancing ageing of the population and the combined effects of Western lifestyles-such as high-fat and high-sugar diets, lack of physical activity and obesity-the risk of CRC has remained persistently high. It ranks third in global cancer incidence and second in cancer-related deaths (2). It is projected that by 2050, the number of new CRC cases worldwide will reach 2.36 million, representing a 22.51% increase from the 2022 baseline (3). Owing to the early roll-out of colonoscopy screening programmes and the implementation of intervention strategies, the incidence and mortality rates of CRC have gradually plateaued or shown a marginal downward trend in recent years. In contrast, in low- and middle-HDI countries undergoing rapid economic transition, CRC incidence is rising rapidly due to the unequal distribution of healthcare and preventive resources and a sharp increase in exposure to environmental risk factors (4).

As a recognised core precancerous lesion of CRC, CRA exhibits a high incidence of occult cases and a clear tendency towards malignant transformation in individuals aged 50 and over. Precise identification at the adenoma stage, supplemented by endoscopic polypectomy, is currently the most effective secondary prevention strategy for reducing the incidence of CRC in the population and optimising long-term survival rates (5).

Consequently, establishing a risk stratification mechanism to accurately distinguish adenomatous from non-neoplastic polyps in patients already scheduled for or undergoing endoscopy not only helps to significantly reduce the loss of disability-adjusted life years (DALYs) and direct healthcare expenditure, but also holds profound clinical significance for optimising endoscopic triage and prioritising pathological evaluation within the cohort (6, 7).This study aims to investigate in depth the association between routine and cost-effective metabolic and inflammatory parameters-such as the peripheral blood TyG index and NLR-and the risk of developing CRA. Building on these findings, we will integrate various objective laboratory indicators to develop a personalised nomogram-based clinical risk prediction model, thereby providing a simple, non-invasive and valuable tool for distinguishing adenomatous from non-neoplastic polyps in patients already scheduled for or undergoing endoscopy.

## 2 Materials and methods

### 2.1 Study population and grouping

#### 2.1.1 Study population

Using the Hospital Information System (HIS) of the Affiliated Hospital of Shandong Second Medical University, patients with colorectal polyps who underwent virtual colonoscopy and successful endoscopic resection between 1 January 2020 and 31 December 2025 were selected. Cases with incomplete data, those not meeting the inclusion criteria, and those meeting the exclusion criteria were excluded, resulting in a final study population of 2,437 patients. The data were stratified and randomly divided into a training set (n=1,707) and a validation set (n=730) in a 7:3 ratio. Based on histopathological diagnostic criteria, the subjects were classified into a colorectal adenoma (CRA) group (training set: n=1,109; validation set: n=475) and a non-neoplastic polyp group (training set: n=598; validation set: n=255).

This study protocol strictly adheres to the principles of the Declaration of Helsinki and has been reviewed and approved by the hospital’s Medical Ethics Committee (approval number: [SDSMU-2026-ky-069]).

### 2.2 Clinical data

#### 2.2.1 Baseline data

This study systematically collected demographic characteristics (age, gender), lifestyle habits (smoking and alcohol consumption history) and peripheral blood biochemical parameters from the study participants, and used these to calculate derived metabolic and immunological indices such as the SII, NLR and TyG Index.

#### 2.2.2 Calculation of derived indices

This study introduces several derived indices reflecting systemic inflammation and metabolic status in the body. Their specific definitions and calculation formulas are as follows:

Systemic Immune-Inflammation Index (SII):

SII = (Neutrophil count × Platelet count)/Lymphocyte count.

Neutrophil-to-Lymphocyte Ratio (NLR):

NLR = Neutrophil count/Lymphocyte count

Platelet-to-Lymphocyte Ratio (PLR):

PLR = Platelet count/Lymphocyte count

Triglyceride-glucose index (TyG):

TyG = ln(triglycerides × fasting plasma glucose/2)

Note: The units for TG and FPG in the formula are mg/dL.

### 2.3 Statistical methods

All data cleaning, statistical analysis and graphical representation in this study were performed using R software (version 4.5.2) and its associated packages. A single-factor logistic regression model was used to preliminarily identify statistically significant potential risk factors, and these variables were then incorporated into a multivariate logistic regression model to determine the independent risk factors for the development of colorectal adenomas.

Feature dimensionality reduction is performed using LASSO regression. The optimal penalty coefficient λ is determined via 10-fold cross-validation, and the features corresponding to non-zero coefficients are extracted to construct the final predictive model. And we employed the Random Forest algorithm to assess feature importance across the entire cohort.

This study conducted a comprehensive internal validation of the cut-off chart and an internal hold-out validation set using a validation set, based on three dimensions: discriminatory power, calibration, and clinical utility. With regard to clinical utility, decision curve analysis (DCA) was employed to assess the clinical net benefit of the cut-off chart at different risk thresholds.

All hypothesis tests in this study were two-tailed, with a p-value of <0.05 considered statistically significant.

## 3 Results

### 3.1 Characteristics of the study population

This study included patients with colorectal polyps who were admitted to our hospital between January 2020 and December 2025, underwent a virtual colonoscopy, and successfully underwent endoscopic polypectomy. Of these, 10 cases were excluded due to age < 18 years, 35 were excluded due to severe cardiopulmonary dysfunction (coronary heart disease, heart failure, chronic obstructive pulmonary disease, etc.), 53 were excluded due to concomitant malignant tumours, 5 were excluded due to a history of inflammatory bowel disease, and 3 were excluded due to concomitant genetic disorders; ultimately, 2,437 patients were included. There were 1,323 males (54.29%) and 1,114 females (45.71%). The age range of the study population was 18–89 years, with a mean age of 58.57 years and a median age of 60 years. The final cohort was stratified and randomly divided in a 7:3 ratio into a training set (n=1,707) and a validation set (n=730). Based on histopathological diagnostic criteria, the study subjects were classified into the CRA group (training set n=1,109, validation set n=475) and the non-neoplastic polyp group (training set n=598, validation set n=255). See **Figure 1**.

**Figure 1:**
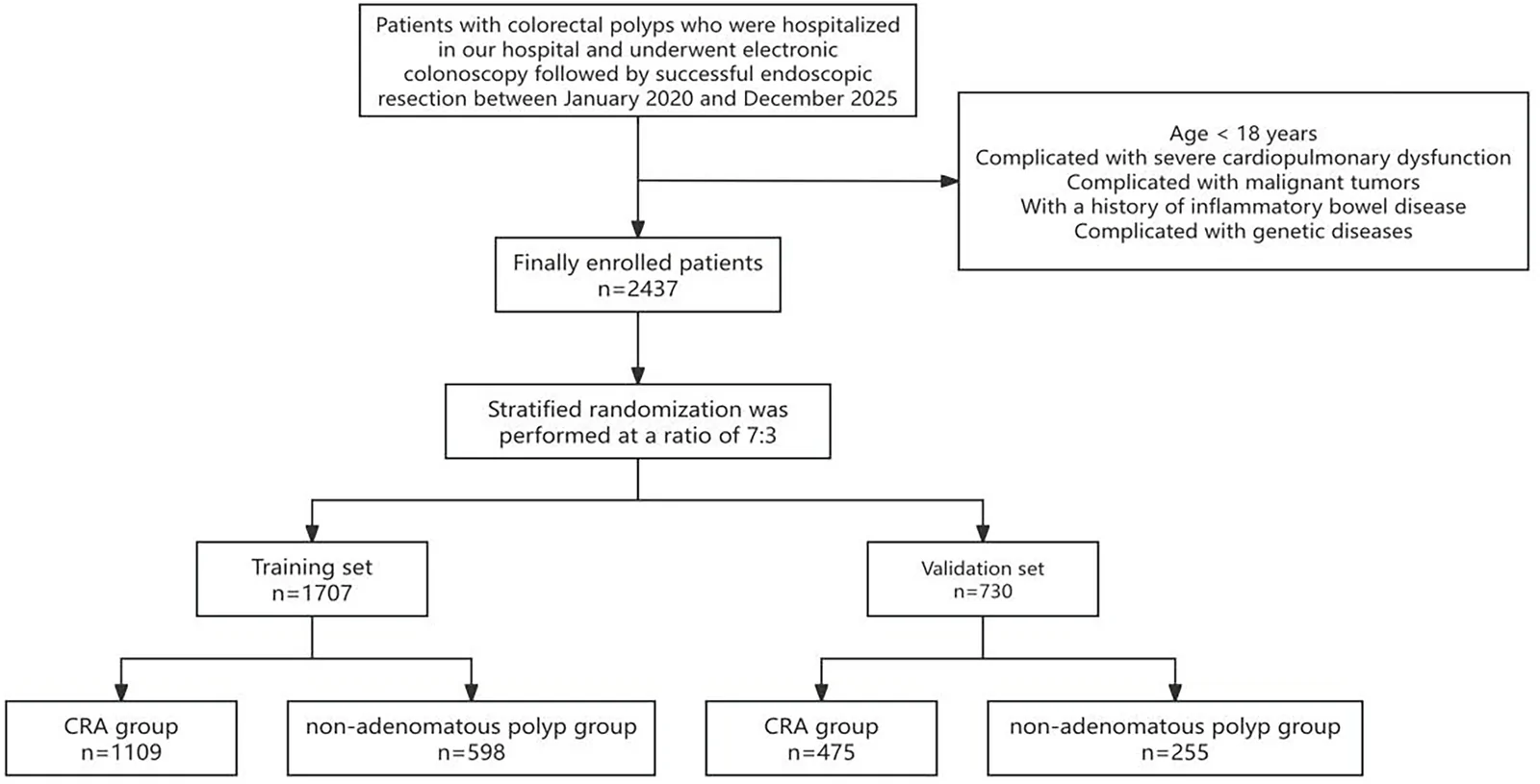
Flowchart of the case selection process.

### 3.2 Comparison of baseline data

In terms of demographic and lifestyle characteristics, the median age in the adenoma group was significantly higher than that in the control group, and the proportion of men was higher. In the adenoma group, the proportion of patients with a history of smoking and alcohol consumption was significantly higher than in the control group. TyG and FPG levels were significantly higher than in the control group. See **Table 1**.

**Table 1:** Characteristics between the adenoma group and the control group.

Variables	Adenoma group (n=1109)	Control group (n=598)	*P*
Age	60.00 [54.00,67.00]	57.00 [49.00,63.00]	<0.001*
Gender			
Male n (%)	654 (59.0)	276 (46.2)	<0.001*
Female n (%)	455 (41.0)	322 (53.8)	
Smoking n (%)			
None	841 (75.8)	497 (83.1)	
Yes	268 (24.2)	101 (16.9)	=0.001*
Drinking (%)			
None	862 (77.7)	490 (81.9)	
Yes	247 (22.3)	108 (18.1)	0.047*
FPG (mmol/L)	5.20 [4.81,5.93]	5.14 [4.69,5.73]	0.006*
HDL-C (mmol/L)	1.28 [1.09,1.51]	1.32 [1.10,1.55]	0.069
LDL-C (mmol/L)	2.99 [2.38,3.60]	2.97 [2.43,3.54]	0.724
TC (mmol/L)	4.87 [4.15,5.61]	4.88 [4.23,5.62]	0.618
TyG Index	8.58 [8.19,8.98]	8.49 [8.15,8.91]	0.019*
NLR	1.89 [1.39,2.62]	1.82 [1.38,2.45]	0.211
SII	404.68 [284.8,588.2]	391.31 [291.7,559.8]	0.440
PLR	126.15 [100.0,159.4]	125.23 [100.0, 162.2]	0.973

P<0.05 indicates a statistically significant difference; continuous variables are expressed as interquartile ranges [Q1, Q3], and categorical variables are expressed as the number of cases (percentage) n (%). * denotes P<0.05 (statistically significant).

### 3.3 Screening of continuous variables and determination of cut-off values

To avoid the influence of outliers on continuous variables in multivariate analysis, this study calculated the optimal cut-off values for continuous variables based on receiver operating characteristic (ROC) curves and the maximum Youden index. The results of the analysis show that the optimal cut-off values for each continuous variable in distinguishing the risk of colorectal adenomas, along with the corresponding area under the curve (AUC), sensitivity and specificity, are as follows (See **Supplementary eTable 1**).

### 3.4 Results of the analysis of independent risk factors

The results of univariate logistic regression analysis showed that being male, age ≥56.5 years, a history of smoking, a history of alcohol consumption, TyG ≥8.562, NLR ≥1.489, fasting blood glucose ≥4.875 mmol/L, and HDL-C <1.315 mmol/L were all significantly associated with the risk of developing colorectal adenomas (all P < 0.05). Detailed results of the univariate logistic regression analysis are presented in **Supplementary eTable 2**.

The results of the multivariate logistic regression analysis showed that male gender (OR = 1.621, 95% CI: 1.269–2.074), age ≥ 56.5 years (OR = 1.868, 95% CI: 1.515-2.205) and TyG ≥ 8.562 (OR = 1.271, 95% CI: 1.015-1.590) were independent risk factors for the development of colorectal adenomas (all P < 0.05). See **Supplementary eTable 3** and **Figure 2**.

**Figure 2:**
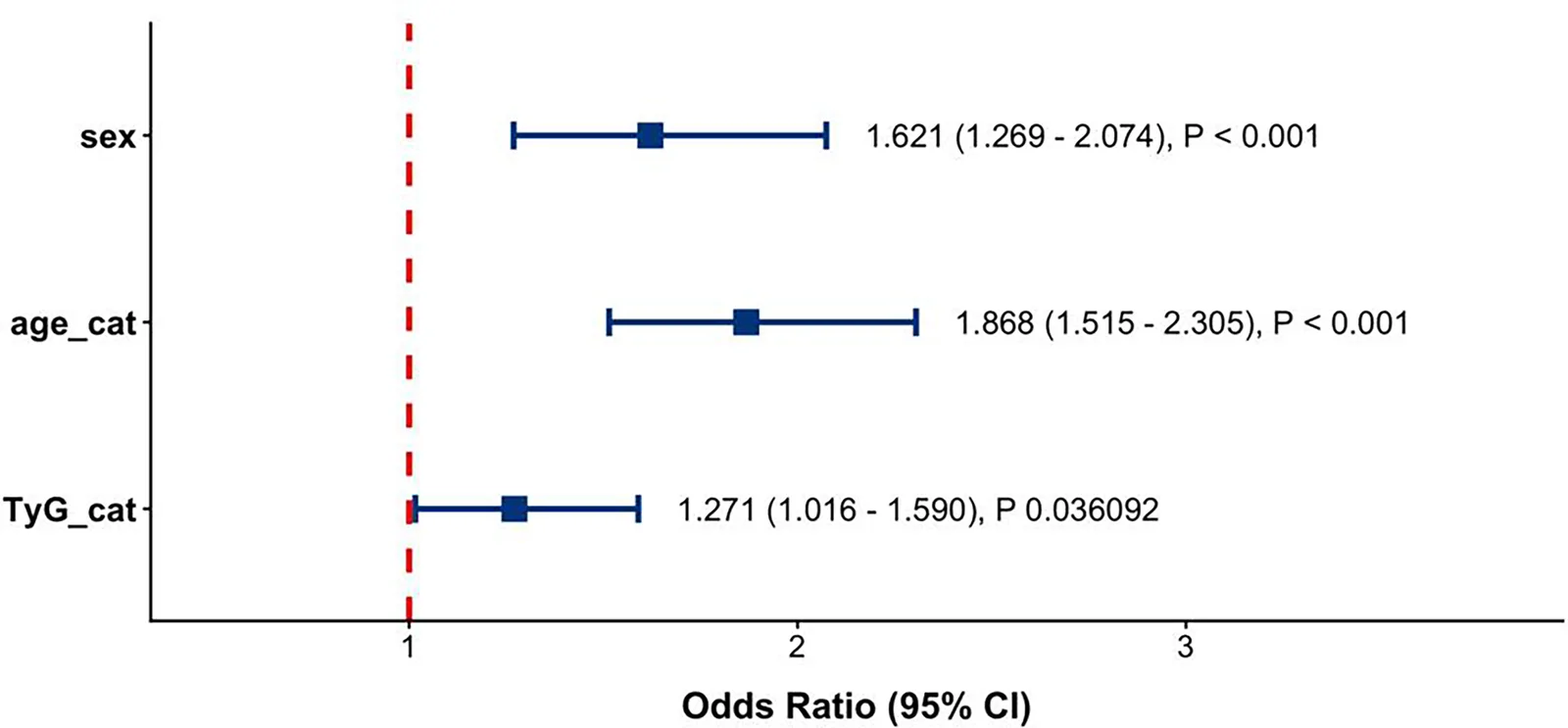
Forest plot of independent risk factors.

### 3.5 Construction of the predictive model

To construct a risk prediction model that retains more comprehensive data information, this study incorporated data as continuous variables in the LASSO regression analysis. The regularisation parameter was optimised using 10-fold cross-validation, ultimately yielding five non-zero coefficient features: gender, age, smoking history, LDL-C and TyG. This study selected -log(λ) as the optimal regularisation parameter; under this parameter, the model incorporated five features, effectively controlling the risk of overfitting whilst ensuring predictive performance. In the LASSO regression analysis, all clinical baseline characteristics and derived variables were included in the screening process. Through 10-fold cross-validation, the bivariate bias curve was plotted against variations in the regularisation coefficient λ. When the bivariate bias reached its minimum, the optimal regularisation parameter λ = 0.00935 was determined, corresponding to log(λ) = -4.67213 (here, the natural logarithm), as shown in **Supplementary eFigure 1**. Based on the selection of -log(λ) = 4.67213, the five features ultimately included in the model correspond to the curves that remain non-zero at this parameter, as shown in **Supplementary eFigure 2**.

Based on these five key characteristics, this study developed a predictive model to estimate the risk of developing colorectal adenomas and produced a scatter plot. In this risk plot, the different categorical states of each independent risk factor are mapped to specific scores; the total score (Total points) is obtained by summing the scores corresponding to each risk factor for an individual, and this corresponds to the predicted probability of the individual developing colorectal adenomas on the risk axis at the bottom of the model, as shown in **Figure 3**.

**Figure 3:**
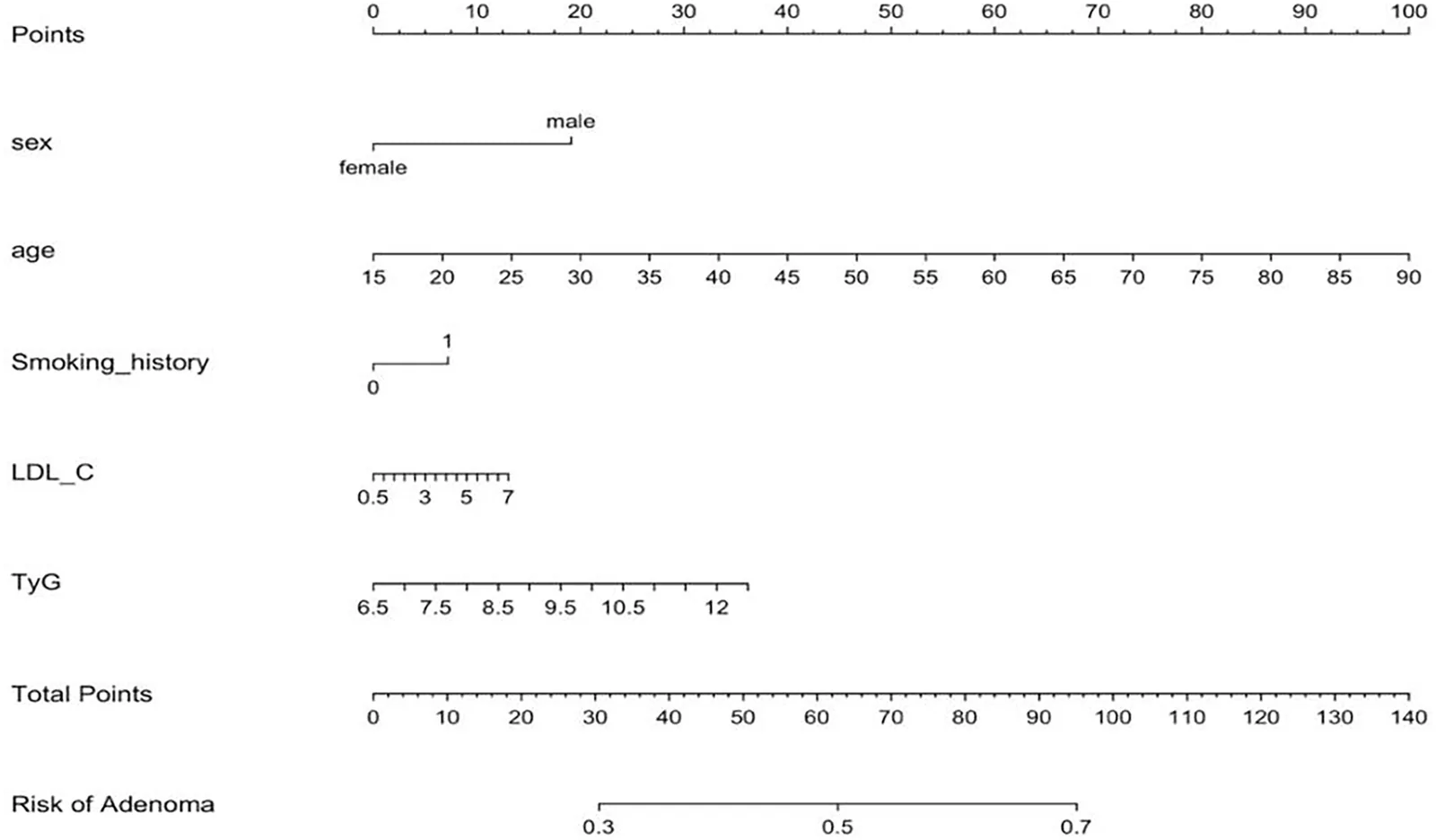
Line graph model for predicting the risk of developing colorectal adenomas.

To use the prediction model, locate the specific variable status for each individual on the axes corresponding to the respective predictors, then trace vertically upwards to the “Points” axis at the top to read the score for that variable. Add the scores for these five indicators to obtain the total score. Locate this value on the ‘Total points’ axis, then trace vertically downwards to the prediction axis at the bottom to determine the patient’s predicted probability of having colorectal adenoma.

### 3.6 Feature importance analysis using random

To further evaluate and test the significance of each predictor for the risk of developing colorectal adenomas, a Random Forest algorithm was additionally applied to rank the relative importance of each predictor from a non-linear perspective. The model comprised 500 decision trees, and the importance of features was assessed using the Mean Decrease Gini.

The Random Forest algorithm was used to evaluate the characteristics of the included clinical and laboratory indicators. The results showed that the predictors included in the predictive model were, in order of importance: age (Mean Decrease Gini = 89.83), TyG (86.22), LDL-C (76.92), gender (13.19) and smoking history (8.18), as shown in **Supplementary eFigure 3**. The x-axis represents the Mean Decrease Gini; a higher value indicates a greater contribution and importance of the corresponding variable to the model’s classification accuracy.

The results of feature importance ranking in the Random Forest model were consistent with the independent risk factors identified by LASSO regression analysis (age, TyG, LDL-C). This indicates that these core indicators demonstrate consistent and stable predictive performance, whether in traditional linear models or in machine learning algorithms designed to handle complex non-linear relationships. The consistency of the results obtained from these two methodologically distinct analytical approaches provides mutually corroborating and supportive evidence for the validity of the aforementioned predictive variables. It should be noted that the importance rankings derived from the Random Forest model merely reflect the contribution of each variable to reducing impurity within that model; they do not constitute an independent statistical validation of the LASSO-based predictive model. Formal validation of the model’s performance (discriminatory power, calibration and clinical utility) will be discussed separately in Section 2.7. However, the predictors for categorical variables-smoking history and gender-exhibited lower importance. This may be due to the tree-based model algorithm’s inherent preference for continuous variables.

### 3.7 Evaluation of model performance

To conduct a comprehensive evaluation of the developed nomogram for predicting the risk of colorectal adenoma, this study utilised the training set for internal validation and the validation set for internal hold-out validation set. The model for predicting the risk of colorectal adenoma was systematically evaluated in terms of discriminatory power, calibration, and clinical utility.

Regarding discriminatory power, the predictive model constructed using factors selected via the LASSO regression algorithm achieved an area under the receiver operating characteristic (ROC) curve (AUC) of 0.631 in the training set and 0.607 in the validation set. This suggests that the nomogram possesses a certain degree of discriminatory power and can distinguish between colorectal adenomas and inflammatory polyps to some extent, See **Figure 4**.

**Figure 4:**
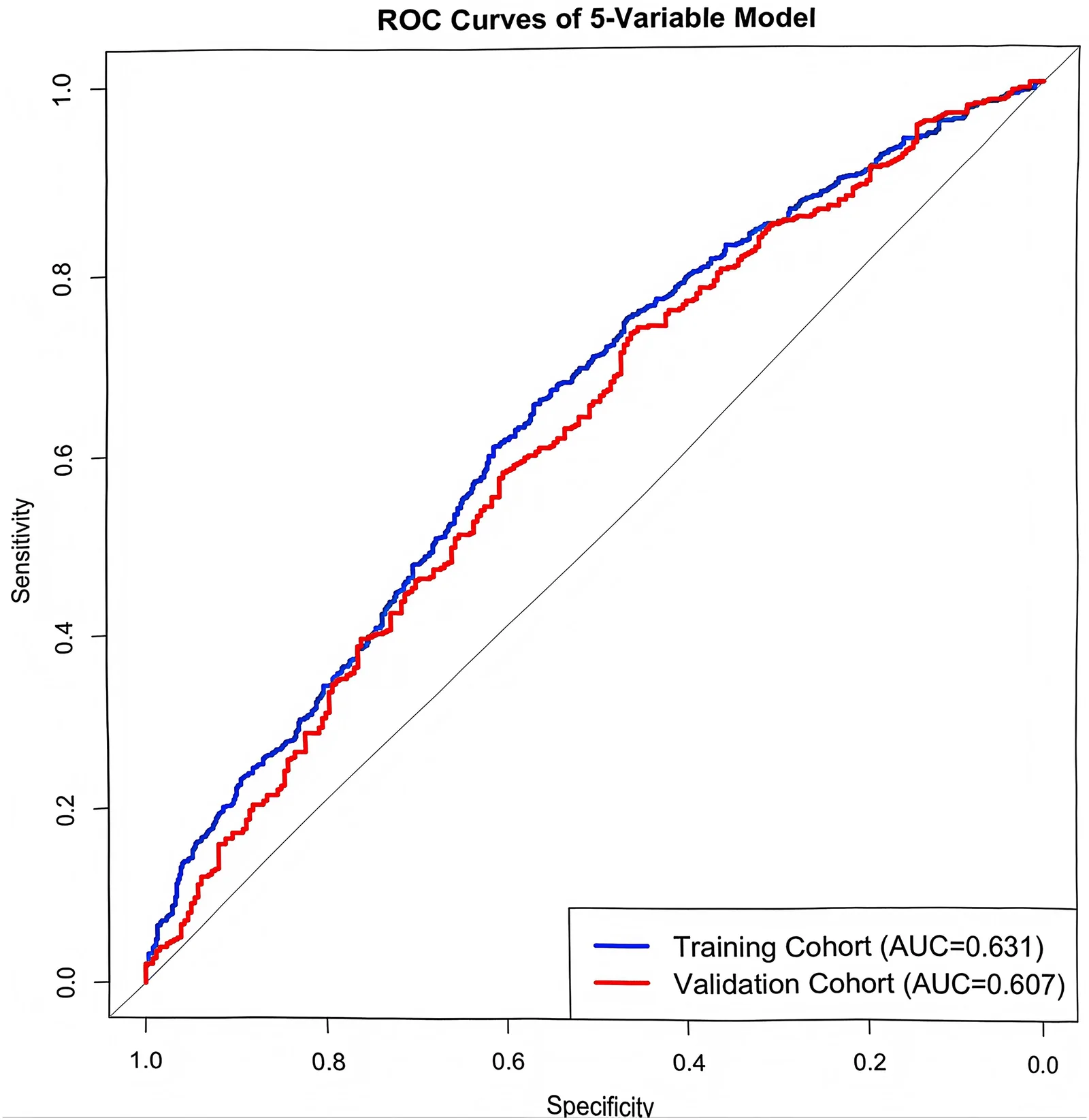
Characteristic curve of the model for predicting the risk of colorectal adenoma.

In terms of calibration, the validation calibration curve plotted using the Bootstrap method (with 1,000 resamples) shows that the probability of colorectal adenoma occurrence predicted by the log-linear model closely follows the diagonal reference line, demonstrating good consistency. There was no significant over- or under-prediction, and the trends of the curves for the training and validation sets were relatively consistent, proving the reliability of the model. The calibration curves (Apparent and Bias-corrected lines) in the figure align closely with the diagonal dotted line, indicating that the model possesses good calibration; the predicted probabilities correspond well with the actual clinical incidence rates, with no significant over or underestimation. See **Figure 5A**.

**Figure 5:**
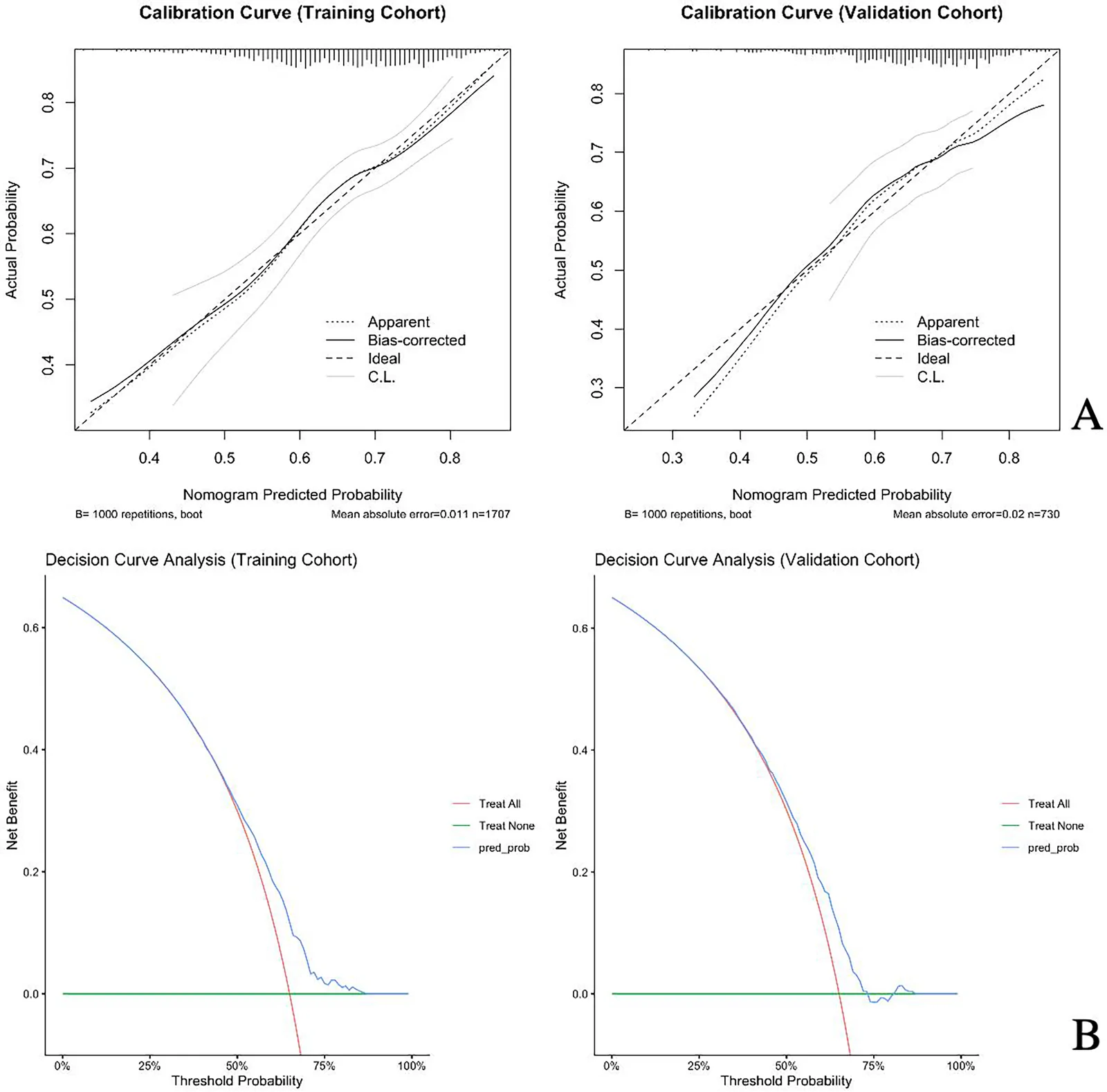
Panel **(A)** calibration curve for the line graph predicting the risk of colorectal adenoma. Panel **(B)** DCA of the line-graph model.

Decision curve analysis (DCA) results indicate that, within a broad range of predicted probability thresholds, the application of this decision tree model to assess the risk of colorectal adenomas and to guide clinical decisions such as whether to prioritise pathological evaluation of the resected polyps, compared to the extreme strategies of “intervening in all patients” or “not intervening in any patients”, yields a superior clinical net benefit for study subjects. This confirms the model’s preliminary reference value in real-world clinical settings, and the relative consistency of the curves between the training and validation sets demonstrates the model’s reliability. The horizontal axis of the figure represents the threshold probability (i.e. the intervention risk threshold acceptable to both patients and clinicians), whilst the vertical axis represents the clinical net benefit. The two extreme reference lines in the figure represent: the “All” line, which assumes that all study subjects develop adenomas (i.e., pathological evaluation is prioritised for every patient); and the “None” line, which assumes that none of the study subjects develop adenomas (i.e., no intervention is performed on anyone). See **Figure 5B**.

## 4 Discussion

Colorectal adenomas are common precancerous lesions in the digestive system; they exhibit a persistently high incidence at the epidemiological level, and their malignant potential has been confirmed in numerous large-scale clinical studies (8). The accurate identification and management of this high-burden pathological entity constitute a core clinical challenge faced by gastroenterologists and general practitioners in the practice of colorectal cancer screening (9). Through early assessment of the pathological nature of polyps and the implementation of precise endoscopic resection, the ‘adenoma-adenocarcinoma’ progression sequence can be effectively interrupted. This holds critical preventive and control value in optimising the long-term survival prognosis of study subjects and enhancing risk stratification within the endoscopic cohort. Against the backdrop of an accelerating ageing population, systemic physiological abnormalities-such as dysregulation of glucose and lipid metabolism-prevalent among the elderly may provide a potential catalytic impetus for the development and malignant transformation of adenomas by reshaping the local intestinal microenvironment. This phenomenon highlights the necessity of incorporating metabolic risk assessment into the risk stratification of patients undergoing endoscopic evaluation.

From the perspective of demographic characteristics and lifestyle, male gender, advanced age particularly those over 50 years of age, and a history of long-term smoking and alcohol abuse have all been identified as independent risk factors for the development of CRA (10). This pathological process is often accompanied by systemic metabolic dysregulation. Our findings indicate that, among the various baseline characteristics and derived indicators included in the analysis, male sex, age ≥56.5 years, and a TyG index ≥8.562 are independent risk factors for the development of colorectal adenomas. Furthermore, by constructing a logistic regression model, it was found that combining the aforementioned demographic characteristics with metabolic and inflammatory derived indicators holds some predictive value for the risk of colorectal adenoma. The area under the receiver operating characteristic (ROC) curve (AUC) was 0.631 in the training set and 0.607 in the validation set.

Building upon the multivariate regression analysis, the Random Forest algorithm was employed to analyse variable importance from a non-linear perspective. Based on the average reduction in the Gini coefficient, age, sex, the triglyceride–glucose index (TyG) and the neutrophil-lymphocyte ratio (NLR) were all identified as variables of high importance; this is consistent with the results of the variables retained following multivariate logistic regression. The fact that the results from these two analytical methods, which are based on fundamentally different principles, are consistent with one another enhances the credibility of the association between these variables; however, this does not constitute formal model validation, nor does it rule out other potential sources of bias, such as unmeasured confounding factors. In addition to statistical efficacy, this study evaluated clinical utility using DCA. Across a wide range of threshold probabilities, the model provided a positive net benefit relative to the treat-all and treat-none strategies, although this should be interpreted in light of the model’s modest discriminatory performance (AUC 0.607-0.631). Taken together, these findings highlight the clinical necessity of integrating assessments of systemic inflammation and metabolic burden. This comprehensive assessment approach can assist clinicians in accurately stratifying high-risk groups for CRA among patients undergoing endoscopic evaluation, and offers clear guidance for optimising existing endoscopic triage strategies and prioritising pathological evaluation. Collectively, the combination of LASSO, Random Forest, nomogram, and DCA provides internally consistent, hypothesis-generating evidence for these predictors. Given that the model’s AUC value is only moderate and the data were derived from a single-centre retrospective study, the statistical performance and clinical utility of this model should be regarded as preliminary findings only and require confirmation through external prospective validation.

### 4.1 Risk factors associated with colorectal adenomas

#### 4.1.1 Age and the risk of colorectal adenomas

As the most influential independent risk factor in the development of CRA (11), the pathogenic weight of age exhibits a significant non-linear increase after the age of 50. It is worth noting that the prevalence of early-onset colorectal adenomas (EOCRA) among individuals under the age of 50 has increased in recent years (12), and this shift in the epidemiological pattern suggests that the clinical risk of onset is evolving from a traditional single-peak distribution towards a potential bimodal pattern. The empirical data from this study are highly consistent with the aforementioned trend. Multivariate analysis confirmed that the risk of developing adenomas in those aged ≥56.5 years was 1.868 times higher than in younger age groups (OR = 1.868, 95% CI: 1.515-2.205, P < 0.001). This finding was further corroborated by the Random Forest analysis, which independently identified age as the most important predictor through non-linear association. The results showed that the feature importance weight for age, based on the average reduction in the Gini index, reached 89.83, ranking first among all candidate predictor variables.

The incidence of early-onset colorectal cancer has shown a marked upward trend in recent years, making risk assessment in young adults aged 40 and under increasingly important. A comparative study involving 805 young participants indicated that the incidence of CRA in this age group is rising rapidly in tandem with an increase in modifiable metabolic factors and lifestyle exposures (13). Another cross-sectional study of adults aged 20–49 also clearly indicated that young-onset colorectal adenomas (YOAs) have become a major risk factor for the development of early-stage CRC. This phenomenon suggests that the occurrence of YOAs is not an isolated pathological event, but rather is deeply functionally coupled with the body’s systemic metabolic health status (14).

The age-risk distribution patterns revealed in this study are highly consistent with the previous consensus on the dual-tiered effect of CRA incidence, thereby reaffirming at a single-centre level that individuals aged 50 and over constitute a high-risk group for colorectal cancer. The optimal age cut-off identified in this study (56.5 years) differs from the findings of cross-sectional studies that have focussed on younger adults under the age of 40. This heterogeneity may be attributed to the geographical distribution of our sample, the composition of the single-centre cohort, and differences in the sensitivity of inclusion and exclusion criteria when screening populations with varying risk profiles; collectively, these factors have led to the age effect manifesting to varying degrees across different research settings.

#### 4.1.2 Gender and the risk of colorectal adenomas

Male gender is considered a robust predictor of the risk of developing colorectal adenomas (CRAs) and subsequent colorectal cancer (CRC) (15). In the data from this study, the proportion of men in the CRA group was higher than in the control group,654 (59.0%) vs. 276 (46.2%), P < 0.001; multivariate logistic regression analysis further confirmed that male sex is an independent risk factor for the development of colorectal adenomas (OR = 1.621, 95% CI: 1.269-2.074).

Global epidemiological surveillance data clearly illustrate significant differences in the gender distribution of colorectal cancer. Statistics show that CRC ranks third among malignant tumours in men, with a global age-standardised incidence rate (ASIR) of 23.4 per 100,000, which is significantly higher than the rate of 16.2 per 100,000 observed in women (16). This macro-epidemiological trend is also reflected at the micro-level in clinical screening practice. In a cross-sectional study involving 2,042 participants, the detection rate of CRA among male participants reached 54.2 per cent, which was considerably higher than the 30.9 per cent observed among female participants; this difference in susceptibility between the sexes was statistically significant (17). Metabolic health status and gender-specific characteristics exert synergistic effects during the progression of the disease. The TyG collaborative study revealed that when men exhibit abnormal metabolic markers, the incidence of multiple colorectal adenomas (≥2 lesions) tends to double. The proportion of male participants detected with advanced adenomas during screening was also higher than that of women in the same age group (18).

The marked susceptibility of men to colorectal cancer is not only attributable to differences in external lifestyle exposures, such as smoking and alcohol consumption, but is also, at a deeper level, driven by the synergistic interaction of complex endocrine homeostasis heterogeneity and chromosomal genetic characteristics. Within this mechanism, imbalances in sex hormone homeostasis, particularly shifts in the oestrogen-to-testosterone ratio (E/T ratio) are regarded as key factors in the development of intestinal lesions. Visceral fat, as a key peripheral site for oestrogen synthesis in men (19), can mediate a progressive decline in endogenous testosterone levels as men age or as abdominal obesity worsens. Concurrently, the aromatase-mediated conversion of androgens to oestrogens is significantly enhanced, ultimately inducing a physiological state characterised by testosterone deficiency and relative oestrogen excess. It is worth noting that, even after adjusting for the confounding factor of body mass index (BMI), a high E/T ratio was still found to be directly associated with the risk of colorectal cancer in men (20). The sex-specific nature of gene expression profiles and molecular phenotypes also plays a decisive role in shaping tumour progression pathways. An in-depth analysis of the Cancer Genome Atlas confirmed that approximately 87.7 per cent of the genes exhibiting sex-specific expression in colorectal cancer are located on the sex chromosomes (X or Y) (21).

#### 4.1.3 Tobacco exposure and the risk of colorectal adenomas

Statistical analysis of the baseline data from this study showed that the proportion of patients with a history of smoking was significantly higher in the CRA group than in the control group (24.2% vs. 16.9%, P = 0.001). An *in vivo* exposure experiment conducted using CPC-APC mice (a human analogue model of familial adenomatous polyposis carrying a mutation in the APC gene, which typically develops spontaneous colonic adenomas within 16 weeks) observed that, compared with the control group breathing clean air, mice in the experimental group that were chronically exposed to conventional cigarette smoke or e-cigarette aerosol showed a significant upward trend in both colon polyp burden and microscopic pathological malignancy scores compared with the control group breathing clean air (22). A meta-analysis incorporating 188 studies further confirmed a robust linear positive correlation between cumulative smoking volume (pack-years) and the risk of colorectal cancer (CRC) (23).

The findings of this study further confirm the robust association between tobacco exposure and the risk of developing CRA. The results of this study are highly consistent with those of animal exposure experiments conducted under a genetic mutation model. However, as this is a retrospective study, although differences were observed in univariate analysis, these were not incorporated into the independent risk factor model in the multivariate logistic regression. This may be due to sample size, information bias or multicollinearity with other metabolic markers.

The pathological mechanism by which tobacco causes CRA is not merely the systemic dissemination of toxins via the systemic circulation, but rather relies on the remodelling of the local intestinal microenvironment to trigger a complex carcinogenic process. From a molecular perspective, reactive oxygen species and polycyclic aromatic hydrocarbon carcinogens produced by tobacco combustion systematically disrupt the body’s antioxidant defence system. This imbalance not only triggers a lipid peroxidation cascade, leading to the production of cytotoxic aldehyde intermediates, but also causes stable DNA adducts to form in intestinal epithelial cells, thereby inducing mutations at key sites of tumour suppressor genes and acting as a trigger for the transformation of early-stage adenomas into malignant phenotypes (24). The accumulation of genetic damage is limited by a decline in the cells’ repair capacity. Research findings indicate that tobacco exposure can specifically suppress the expression of key DNA repair enzymes, such as NEIL-2 and OGG1, in colonic cells, preventing tobacco-induced base damage from being accurately repaired and thereby increasing genomic instability. Concurrently, the transcriptional levels of CD44 and Lgr-5 markers of tumour stem cells at the base of colonic crypts rise significantly in response to tobacco stimulation. This phenotypic shift confers strong anti-apoptotic properties to mutated cells, promoting the clonal proliferation of micro-polyps at the microscopic level (22). Concurrently, the intestinal phospholipase D pathway undergoes abnormal activation. Dysregulation of this pathway not only exacerbates local inflammatory infiltration and oxidative stress but also alters metabolic products, creating a favourable niche for the colonisation of oncogenic pathogens such as Fusobacterium nucleatum. Tobacco metabolites disrupt microbial homeostasis, promoting the production of secondary oncogenic metabolites and accelerating the malignant progression of the intestinal mucosa (25).

#### 4.1.4 LDL-C, HDL-C and the risk of colorectal adenomas

LDL-C has been demonstrated to be a risk factor for the development of colorectal adenomas (CRA), whilst HDL-C levels have been shown to exhibit a significant inverse correlation with the risk of CRA development (26). Regrettably, this study did not establish that LDL-C and HDL-C are independent risk factors for colorectal adenomas. The predictive value of LDL-C and HDL-C for colorectal adenomas was only demonstrated in univariate regression analyses and in the exploratory Random Forest importance ranking.

Studies have shown that LDL-C levels are significantly associated with the presence and number of colorectal polyps (P < 0.05) and may serve as a predictor of polyp risk (27). A Mendelian randomisation study has also indicated that LDL-C is an independent risk factor for colorectal adenomas (28). Previous clinical controlled studies have revealed (29) that peripheral blood HDL-C levels in patients with colorectal adenomas were significantly lower than those in healthy control groups (P < 0.001). Furthermore, a meta-analysis incorporating 37 clinical studies, comprising a total of 19,464 cases of colorectal polyps and 63,979 controls, confirmed that HDL-C levels in patients with colorectal adenomatous polyps were significantly lower than those in healthy controls (weighted mean difference, WMD = -2.589 mg/dL, 95% CI) (30).

#### 4.1.5 Triglyceride-glucose index and the risk of colorectal adenomas

An abnormally elevated triglyceride-glucose index (TyGIndex) not only shows a clear positive association with the initial risk of colorectal adenoma (CRA), but has also been identified as a key predictor of recurrence following polypectomy, the formation of multiple lesions, and progression to advanced adenomas. As an assessment tool that integrates information on lipid metabolism and glucose homeostasis, the TyG Index is considered to be more sensitive than traditional single fasting blood glucose measures in reflecting metabolic disorders (31). This observation was confirmed in the present study. In the baseline analysis, the TyG index was significantly higher in the study group than in the control group [8.58 (8.19, 8.98) vs. 8.49 (8.15, 8.91), P = 0.019]. Multivariate modelling ultimately confirmed that a TyG index ≥ 8.562 is a key independent risk factor for the development of colorectal adenomas (OR = 1.271, 95% CI: 1.015-1.590, P = 0.003).A multivariate logistic regression analysis clearly indicated that an abnormally elevated TyG index is an independent risk factor for the development of colorectal adenomas and postoperative recurrence; compared with healthy individuals in the lowest quartile of the TyG index, those in the highest quartile had a 5.079-fold increased risk of developing multiple adenomas (≥2 in number) (20). The TyG index is an independent risk factor for adenoma development and recurrence (OR = 1.494) (32).

Our findings show a high degree of consistency with the observations from numerous large-scale clinical cohort studies both in China and internationally. This conclusion not only corroborates the view, established in previous studies, that pathologically elevated TyG indices significantly increase the risk of adenoma development and post-operative recurrence, but also provides strong mutual validation of domestic evaluations indicating that this index possesses good predictive performance (AUC = 0.756). The risk effect estimate obtained in this study (OR = 1.271) appears to differ in magnitude from some previous findings in studies of subjects with multiple adenomas (where the risk was increased by up to 5.079-fold); This discrepancy may be attributed to the fact that the cohort in this study comprehensively encompassed both solitary and low-risk adenoma groups, whilst objective heterogeneity exists across different studies in terms of geographical distribution characteristics, criteria for establishing cut-off values, and the baseline metabolic profiles of study participants, thereby leading to differences in the weighting of risk predictions.

Elevated TyG indices, indicative of insulin resistance, disrupt the balance between IGF-1 and insulin-like growth factor-binding protein 3 (IGFBP3) in the body, leading to a significant increase in circulating IGF-1 levels and a suppression of IGFBP3 synthesis and activity. IGF-1 is significantly associated with an increased risk of colorectal precancerous lesions (33–35), whilst IGFBP3 attenuates its inhibitory effect on IGF-1 binding (36).

### 4.2 A comparative analysis of this study with previous colorectal adenoma prediction models

In recent years, research into non-invasive colorectal adenoma risk prediction models has become increasingly diverse. In order to objectively evaluate the clinical value and practical applications of the model developed in this study, we have systematically reviewed relevant colorectal adenoma and polyp prediction models published domestically and internationally in recent years, and conducted a comparative analysis across several key aspects, including core input variables, model algorithms, sample size, and predictive performance (AUC). Compared with models relying on subjective questionnaires and traditional clinical features, this study is more objective. For example, the clinical questionnaire model developed by Yin Cao et al. (37) based on a US population had AUCs of 0.60 and 0.67 for women and men, respectively, which are broadly on a par with the results of this study (AUC = 0.631). However, questionnaire-based models are severely limited by patients’ subjective recall bias. Whilst the model by Donglin Li et al. (38) incorporated objective indicators such as thrombin time, it still relied on subjective reports of abnormal bowel movements and a history of polyps. In contrast, this study utilised objective laboratory parameters from peripheral blood (TyG and NLR), which can objectively reflect the body’s real-time metabolic and inflammatory microenvironment.

This study falls significantly short of the performance achieved by state-of-the-art multimodal large language models and high-dimensional testing. Xiaoyu Yang et al. (39) extracted 16 multimodal features, including genetic data, from a dataset of nearly 100,000 electronic medical records (EMR) using the BGE-M3 large language model, achieving an AUC of 0.985. Eskelinen et al. (40), by combining the FIT test with neural network algorithms, also achieved high performance ranging from 0.840 to 0.977. However, the high predictive power of such models relies on expensive genetic testing and substantial computational resources, making their widespread application within primary healthcare systems difficult at this stage. In contrast, this study prioritised clinical practicality: by utilising four routine indicators that are easily obtained without additional costs, combined with one personal habit indicator with minimal recall bias, we achieved an extremely low operational threshold and clinical interpretability through line charts, thereby facilitating practical risk stratification within the endoscopy-referred cohort.

From a methodological perspective regarding model construction, LASSO regression was used to identify predictive features. The use of continuous variables rather than converting them into categorical variables based on empirical normalisation or dichotomisation, reduced the loss of information associated with categorisation. Nevertheless, in future prospective cohort studies with larger sample sizes, more advanced statistical methods could be employed to further investigate the dose-response relationships between the aforementioned inflammatory and metabolic markers and the risk of adenomas.

### 4.3 The value of random forests in machine learning for clinical prediction models

#### 4.3.1 Complementary advantages of traditional linear models and machine learning algorithms

In the process of constructing clinical prediction models, ensuring the robustness of feature selection and thoroughly exploring the complex relationships between variables are key to enhancing the scientific rigour of the model. Traditional logistic regression models offer excellent clinical interpretability, enabling the impact of each risk factor on the risk of CRA to be intuitively reflected through the odds ratio (OR) (41). However, logistic regression and LASSO regression are essentially generalised linear models, which often require strict linear assumptions between variables and have certain limitations when handling high-dimensional data and complex interactions between variables (42). Random forests, as an ensemble algorithm based on decision trees, impose no strict requirements on the distribution characteristics of the data and are capable of capturing potential non-linear relationships and higher-order interaction effects between variables (43). In this study, a forest comprising 500 decision trees (ntree=500) was constructed using multiple bootstrap sampling, thereby effectively reducing the risk of overfitting that might arise from a single decision tree and enhancing the generalisation ability of feature selection.

#### 4.3.2 Clinical implications of variable importance ranking

The average reduction in the Gini coefficient for the predictors included in the predictive model in this study was, in descending order: age (MeanDecreaseGini=89.83), TyG (86.22), LDL-C (76.92), gender (13.19) and smoking history (8.18). Age, TyG and LDL-C play a decisive role in predicting the risk of CRA. In the random forest variable importance assessment, the mean Gini coefficient reduction for gender and smoking history was relatively small. This does not imply that they lack clinical value, but rather stems from the mathematical properties of the tree-based algorithm itself. When splitting nodes, Random Forests naturally favour continuous variables with multiple potential splitting points (age, TyG index and inflammatory markers), whereas binary variables (gender, smoking history), due to their single splitting dimension, are prone to algorithmic bias during the importance evaluation process and are consequently underestimated. However, in LASSO regression and multivariate logistic regression which are based on linear assumptions, both gender and smoking history demonstrated strong independent predictive power. This precisely demonstrates the value of cross-checking findings using both traditional linear statistical models (logistic/LASSO) and non-linear machine learning algorithms (Random Forest) in this study. Linear models accurately identified clinically accessible independent risk factors for classification, whilst machine learning algorithms delved deeper into the non-linear contributions of lipid and glucose metabolism and systemic inflammation markers to the pathogenesis. The combination of these dual perspectives provides extremely comprehensive evidence to support the comprehensive screening of colorectal adenomas.

### 4.4 Model calibration and clinical utility

#### 4.4.1 Calibration

Calibration assesses the consistency between the model’s predicted probabilities and the actual incidence of disease (44). If the model is overfitted, it may result in many low-risk patients undergoing unnecessary invasive colonoscopies, thereby increasing healthcare costs and the incidence of complications. Conversely, if the model underpredicts, it may lead to the missed diagnosis of high-risk adenoma patients. In this study, internal validation was performed using 1,000 bootstrap resamples, yielding results showing that the calibration curves (both Apparent and Bias-corrected lines) of the log-linear model were very close to the diagonal. These results indicate that the predictive model established based on sex, age, smoking history, LDL-C and TyG produces a probability of CRA occurrence that is very close to the true incidence of CRA, demonstrating that the model is reliable in terms of probabilistic assessment and can provide clinicians with a reliable reference standard.

#### 4.4.2 Decision curve

Traditional diagnostic performance metrics (sensitivity and specificity) generally treat false positives and false negatives as equally important; in clinical practice, this is incorrect. The cost of missing a high-risk adenoma (false negative) far outweighs that of subjecting a healthy individual to an unnecessary colonoscopy (false positive). DCA uses a threshold probability to comprehensively balance the benefits and risks of an intervention, thereby evaluating the model’s actual clinical value (45). The DCA results of this study indicate that, within a broad range of threshold probabilities, conducting a CRA risk assessment on study subjects using this nomogram model to guide the decision on whether to prioritise pathological evaluation yields a clinical net benefit that is significantly superior to the extreme “blind” strategies of performing colonoscopy on everyone or taking no action for anyone.

Using the flowchart developed in this study, a feasible triage process can be designed for patients who have booked or are currently undergoing endoscopy: Clinicians collect basic demographic information from patients, such as age and gender, enquire about smoking history, extract key indicators from routine blood test results including LDL-C levels, and rapidly calculate the TyG index. Finally, they use the flowchart to aggregate the scores for each indicator to obtain the final predicted probability. A prediction probability of 40 per cent is used here solely as an example to illustrate how model outputs can be translated into binary clinical decisions; based on the dataset from this study alone, it is not yet possible to recommend a definitive clinical cut-off value, the results of the decision curve analysis show that the model yields a positive net benefit across a wide range of probability values, and does not possess clinical value only at a single, fixed cut-off point. To identify clinically appropriate thresholds, it is necessary to clearly weigh up the relative costs of missing adenomas against those of unnecessary histological examinations, taking full account of the risk preferences of both clinicians and patients, and to validate these findings in external prospective cohorts; until this work has been completed, this stratified triage strategy is not yet recommended for direct application in clinical practice.

## 5 Conclusions

1. Patients with colorectal adenomas differ significantly from those with non-neoplastic polyps in terms of age, proportion of males, history of smoking and alcohol consumption, and TyG and FPG levels.
2. Gender, age and the TyG index are independent risk factors for the development of colorectal adenomas.
3. The risk prediction model based on demographic characteristics and metabolic and inflammatory markers, constructed using a line chart, can assist in distinguishing adenomatous from non-neoplastic polyps among patients undergoing endoscopy, demonstrating a certain degree of discriminatory power, with an AUC of 0.631 in the training set and 0.607 in the validation set, and possesses preliminary predictive value.

## 6 Limitations of the study

Whilst this study yielded the anticipated findings, it remains subject to the following limitations that require urgent attention. Firstly, this study employed a retrospective, single-centre design, with clinical data covering only subjects who underwent colonoscopy and polypectomy at our hospital between 2020 and 2025. Although data with missing laboratory parameters were excluded in accordance with the principle of complete case analysis, this screening process may have resulted in a degree of geographical limitation and selection bias. Secondly, the development of colorectal adenomas is governed by complex biological and environmental interactions. The results of this study may be influenced by certain confounding factors not included in the analysis, such as the subjects’ precise dietary patterns, levels of physical activity, and family history-potential risk factors which may result in residual confounding effects.

As this was a retrospective study, patients’ smoking and alcohol consumption histories were derived from their hospital records. Due to variations in the way doctors document case records, it was not possible to ascertain the extent of patients’ smoking and alcohol consumption. The fact that only binary variables were used for analysis in this study constitutes a limitation of the study.

## 7 Future research directions

Future research could explore the following areas in greater depth:

1. The current study developed a model based on single-centre, retrospective data; although its stability has been demonstrated using an internal validation set, the AUC values suggest there is still considerable room for improvement. Future research should leverage regional healthcare alliances to conduct large-scale, prospective, multi-centre cohort studies across different geographical areas.
2. The predictive indicators in this study primarily focus on peripheral blood biochemical and immunological parameters, and do not yet adequately reflect the local intestinal microenvironment. The gut microbiota and its metabolites (such as short-chain fatty acids and secondary bile acids) play a key role in the development of CRA induced by metabolic disorders. Future model development could consider integrating faecal DNA and RNA testing, gut microbiota abundance scores, and proteomic indicators to enhance the model’s sensitivity in distinguishing colorectal adenomas.
3. With the widespread adoption of electronic health records, future research should focus on utilising deep learning algorithms to process longitudinal medical data. Compared to the static analysis in this study, the fluctuating trends of dynamically monitored indicators may hold greater predictive value. By integrating the line chart model from this study into hospital HIS systems and developing automated risk warning plugins, real-time monitoring and precise triage of individuals at risk can be achieved, thereby promoting early screening and treatment of colorectal cancer.

## Data Availability

The original contributions presented in the study are included in the article/**Supplementary Material**. Further inquiries can be directed to the corresponding author.
