# Supplementary material for: Risk factors associated with colorectal adenomas and the formulation of prediction models
Source: Front Oncol. 2026 Oct 12;16:1894239. doi: 10.3389/fonc.2026.1894239 (PMC13696314; doi:10.3389/fonc.2026.1894239)
Supplement: Supplementary file 1 [file SupplementaryFile1.docx]

| eTable 1 Analysis of the diagnostic performance of the various indicators for adenomas | | | | | | |
| --- | --- | --- | --- | --- | --- | --- |
| Variables | Optimal cutoff value | AUC | Sensitivity | Specificity | Jorden Index | *P* |
| Age | 56.5 | 0.597 | 0.655 | 0.495 | 0.150 | <0.001 |
| FPG | 4.875 | 0.540 | 0.714 | 0.370 | 0.084 | 0.006 |
| TC | 4.175 | 0.507 | 0.259 | 0.776 | 0.035 | 0.618 |
| HDL-C | 1.315 | 0.527 | 0.545 | 0.513 | 0.058 | 0.069 |
| LDL-C | 3.735 | 0.505 | 0.208 | 0.828 | 0.036 | 0.724 |
| TyG | 8.562 | 0.534 | 0.518 | 0.560 | 0.079 | 0.019 |
| NLR | 1.489 | 0.518 | 0.707 | 0.343 | 0.050 | 0.211 |
| PLR | 119.808 | 0.500 | 0.576 | 0.457 | 0.033 | 0.973 |
| SII | 382.668 | 0.511 | 0.558 | 0.487 | 0.045 | 0.440 |

| eTable 2 Univariate logistic regression analysis of the risk of colorectal adenoma | | | | | |
| --- | --- | --- | --- | --- | --- |
| Variables | B | SE | *χ²* | OR（95%CI） | *P* |
| Male | 0.517 | 0.102 | 25.560 | 1.677（1.373-2.050） | <0.001* |
| Age | 0.619 | 0.103 | 35.935 | 1.858（1.518-2.276） | <0.001* |
| Smoking | 0.450 | 0.130 | 12.022 | 1.568（1.220-2.029） | <0.001* |
| Drinking | 0.262 | 0.128 | 4.171 | 1.300（1.013-1.677） | 0.041* |
| FPG | 0.382 | 0.108 | 12.558 | 1.465（1.186-1.808） | <0.001* |
| HDL-C | -0.233 | 0.102 | 5.235 | 0.793（0.649-0.967） | 0.022* |
| LDL-C | 0.235 | 0.131 | 3.200 | 1.264（0.980-1.640） | 0.073 |
| TC | 0.190 | 0.120 | 2.516 | 0.827（0.653-1.044） | 0.112 |
| TyG | 0.316 | 0.102 | 9.599 | 1.372（1.123-1.676） | 0.001* |
| NLR | 0.230 | 0.109 | 4.484 | 1.258（1.017-1.556） | 0.034* |
| PLR | 0.133 | 0.102 | 1.691 | 1.142（0.935-1.395） | 0.193 |
| SII | 0.180 | 0.102 | 3.137 | 1.197（0.981-1.462） | 0.076 |

| eTable 3 Multivariate logistic regression analysis of the risk of colorectal adenoma | | | | | |
| --- | --- | --- | --- | --- | --- |
| Variables | B | SE | *χ²* | OR（95%CI） | *P* |
| Male | 0.483 | 0.125 | 14.858 | 1.621（1.269-2.074） | <0.001* |
| Age | 0.625 | 0.107 | 34.023 | 1.868（1.515-2.305） | <0.001* |
| Smoking | 0.238 | 0.167 | 2.027 | 1.268（0.916-1.764） | 0.154 |
| Drinking | -0.125 | 0.166 | 0.563 | 0.883（0.638-1.223） | 0.453 |
| FPG | 0.174 | 0.118 | 2.182 | 1.190（0.944-1.499） | 0.139 |
| HDL-C | 0.003 | 0.112 | 0.001 | 1.003（0.805-1.251） | 0.977 |
| TyG | 0.240 | 0.114 | 4.393 | 1.271（1.015-1.590） | 0.036* |
| NLR | 0.080 | 0.113 | 0.510 | 1.084（0.868-1.350） | 0.475 |

eFigure 1 The variation of binomial deviance with the regularisation parameter

eFigure 2 Feature coefficient shrinkage path diagram for LASSO regression

Note: The numbers in the figure represent: 1 Male, 2 Age, 3 Smoking history, 4 Alcohol consumption history, 5 FPG, 6 TC, 7 HDL-C, 8 LDL-C, 9 TyG, 10 NLR, 11 PLR, 12 SII.

eFigure 3 Prediction of colorectal adenomas based on the Random Forest algorithm
